# Neonatal neurologic emergencies requiring access to paediatric emergency units: a retrospective observational study

**DOI:** 10.1038/s41598-022-13703-2

**Published:** 2022-06-17

**Authors:** Raffaele Falsaperla, Giovanna Vitaliti, Monica Sciacca, Lucia Tardino, Simona Domenica Marino, Silvia Marino, Carla Moscheo, Mariaclaudia Meli, Maria Concetta Vitaliti, Massimo Barbagallo, Vita Antonella Di Stefano, Marco Andrea Nicola Saporito, Martino Ruggieri

**Affiliations:** 1 Neonatal Intensive Care Unit, Azienda Ospedaliero Universitaria Policlinico “G. Rodolico-San Marco”, San Marco Hospital, University of Catania, Catania, Italy; 2 Pediatrics and Pediatric Emergency Operative Unit, Azienda Ospedaliero Universitaria Policlinico “G. Rodolico-San Marco”, San Marco Hospital, University of Catania, Catania, Italy; 3 Pediatrics Operative Unit, Department of Medical Sciences, Section of Pediatrics, University of Ferrara, Ferrara, Italy; 4 Department of Paediatrics, Azienda Ospedaliera di Rilievo Nazionale e di Alta Specializzazione “Garibaldi”, Catania, Italy; 5 Pediatric General and Acute and Emergency Operative Unit, Cannizzaro Hospital, Catania, Italy; 6 Unit of Rare Diseases of the Nervous System in Childhood, Department of Clinical and Experimental Medicine, Section of Pediatrics and Child Neuropsychiatry, University of Catania, 95124 Catania, Italy

**Keywords:** Health care, Neurology

## Abstract

Herein, authors present a retrospective, multi-center study to determine the number of accesses to Pediatric Emergency Unit (PEU) of patients within 28 days of life, admitted to (1) the Acute and Emergency Pediatric Unit, San Marco University Hospital, Catania, Italy; (2) Garibaldi Hospital for Emergency Care, Catania, Italy; (3) Cannizzaro Hospital for Emergency Care, Catania, Italy. We included neonates admitted for neurologic problems, from January 2015 to December 2020, to the 1—Acute and Emergency Access of the San Marco University Hospital, Catania, Italy [observation center 1 (OC1)]; 2—Garibaldi Hospital for Emergency Care, Catania, Italy (Observation Center 2—OC2); 3—Cannizzaro Hospital for Emergency Care, Catania, Italy (Observation Center 3—OC3). For each patient, we evaluated the severity of urgency, by studying the admission triage-coloured codes, the clinical data at admission and the discharge diagnosis. Neonates who had access to PEU were 812 in the OC1, 3720 in the OC2, and 748 in the OC3 respectively; 69 (8.4%), 138 (3.7%), and 55 (7.4%) was the proportion of neonatal accesses for neurological conditions. We observed that in the study period, the three hospitals had an important decrease of pediatric accesses to their PEU, but the proportion of neonates who had access to the OC1 for neurologic diseases, with respect to the total neonatal accesses, remained stable. We found that the most frequent neurologic disease for which newborns had access to PEU was Cyanosis, (46.1% of all neonatal accesses). Apnea was the second most frequent cause, with a number of 76 accesses (29%). In the literature there are numerous studies on the assessment of diseases that most frequently concern the pediatric patient in an emergency room, but there are very few references on neonatal accesses for urgent neurologic diseases. Therefore, appropriate training is required to avoid unnecessary tests without overlooking potentially serious conditions.

## Introduction

Neonatal neurological emergencies represent a challenge for doctors at the Pediatric Emergency Unit (PEU) because symptoms may be nonspecific. The assessment of neonates in critical conditions requires a deep knowledge of the physiological changes and potentially lethal pathological conditions that can occur at this age.

To date, literature data showed that the most frequently encountered neonatal neurological alterations in PEU are convulsions, lethargy, and coma which can be indicators of purely neurological pathologies as well as organ dysfunctions^1^.

Although non-accidental injury may predispose the infant to acute neurological dysfunction, and should be considered when the anamnestic history is suggestive of injury itself, the non-traumatic conditions remain important etiologic factors for acute neurologic diseases in this age group.

Non-traumatic causes of neurological symptoms are frequent in neonates, prognosis varies and depends on the etiologic factor (organic or not)^1^. Encephalopathy might be one of the causes, and affected infants may present with signs of an overwhelming illness requiring immediate care^2^.

Effective recognition and prompt emergency management of neonatal diseases can be life-saving for these young patients. Appropriate training is required to avoid unnecessary tests without overlooking potentially serious conditions^3^. Therefore, it is necessary to establish a diagnostic protocol, in order to express a rapid judgment on neonatal patients at the access time of the emergency setting and thus reducing potential risks.

Herein, authors present a retrospective, multi-center study to determine the number of accesses to PEU of patients within 28 days of life, admitted to (1) the Acute and Emergency Pediatric Unit, San Marco University Hospital, Catania, Italy; (2) Garibaldi Hospital for Emergency Care, Catania, Italy; (3) Cannizzaro Hospital for Emergency Care, Catania, Italy.

The aim of our study was to analyze the access data of PEUs of the three major hospitals from 2015 to 2020, in order to analyze the percentage of neonates who have access to the emergency room for neurologic diseases.

Our secondary outcomes were to analyze the severity of the pathology of the neurologic neonatal emergency, according to triage-colored codes; moreover, we studied the distribution of neonatal neurologic accesses to the three PEUs analyzed. Finally, the distribution of neurologic disease in the three studied PEUs, evaluating which diseases were the most frequent at each site.

## Materials and methods

Our study was a retrospective multi-center cohort study, from January 2015 to February 2020.

We have thus collected the experience of the three min hospitals in Catania: (1) San Marco University Hospital, Catania, Italy; (2) Garibaldi First Aid Hospital, Catania, Italy; (3) Cannizzaro First Aid Hospital, Catania, Italy.

In our study, we included (1) neonates, aged between 0 and 28 days of life, (2) admitted for neurological problems (head trauma, convulsions, tremors, hypotony, sleep disorders, brachial plexus paralysis, torticollis etc.). We also included neonates with cyanosis and presumably central apnea if no other signs or symptoms were indicative of associated with respiratory disease.

For each patient, we have read all the emergency room reports and any medical records. We evaluated the severity of the disease, by studying the triage-coloured codes of admission, the clinical data at admission and the discharge diagnosis.

We excluded: (1) patients older than 28 days, (2) non-neurological diseases, (3) neonates affected by genetic syndromes.

Clinical information retrieved from the database of the studied patients included anthropometrical and clinical data.

During the clinical, psychic and cognitive evaluation, the following data were collected: familial. Personal, and clinical history; weight; height; evaluation of vital parameters; general and detailed neurologic examination; evaluation of consciousness; presence of comorbidities; eventual drug therapy at the time of first evaluation.

The ethics committee of our institution (University of Catania, Sicily, Italy) approved the study. All research was performed in accordance with relevant guidelines/regulations. Informed consent was obtained from all parents of the studied patients. The research was performed in accordance with the Declaration of Helsinki guidelines^4^.

## Statistical analysis

Statistical analysis was performed using Microsoft Excel 2011 for Mac.

Descriptive statistics were calculated for all clinical variables. Qualitative variables were calculated as percentage and quantitative variables as mean ± standard deviation. Kolmogorov–Smirnov One-Sample Test and statistics were used for testing normal distribution of quantitative variables.

### Ethics approval and consent to participate

The study was approved by the Ethical Committee of the University of Catania, Italy. All parents of the included patients gave their consent to participate to the present study.

### Consent for publication

All parents of the included patients gave their consent for publication of the present data in anonymous form.

## Results

From our data analysis, it emerges that during the study period, from 2015 to 2020, the number of accesses to the included PEUs hospitals was 62.886 for San Marco Hospital (observational centre (OC) 1), 184.224 for Garibaldi (observational centre (OC2) 2), and 53.692 for Cannizzaro (observational centre (OC3) 3) respectively, for a total patient of 300,802.

**Figure 1:**
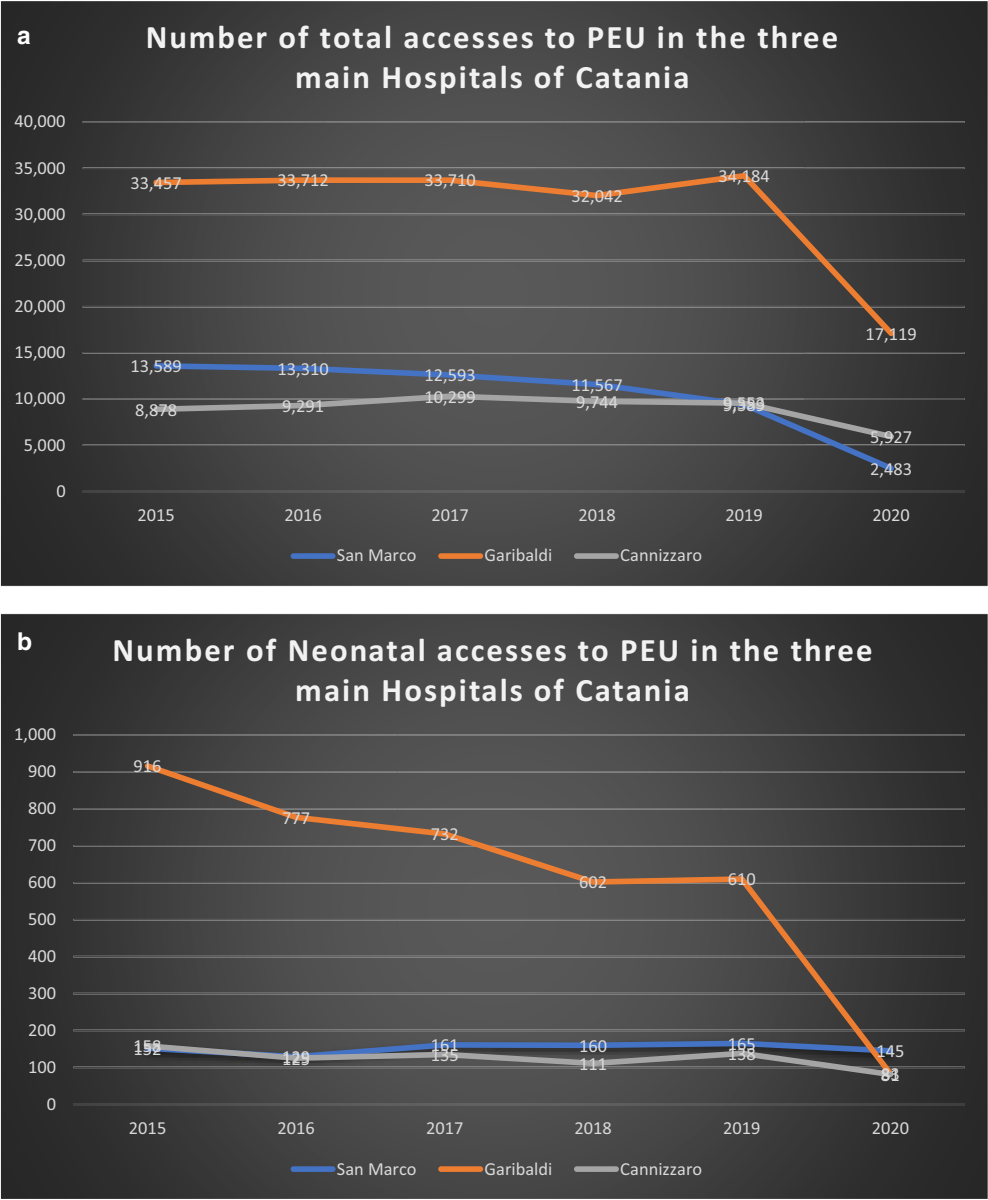
(**A**) Number of total accesses for pediatric emergencies to the PEU of the three main hospitals in Catania. All hospitals had a significative reduction of accesses after SARS-CoV2 Pandemics. (**B**) Number of total neonatal accesses to the PEU of the three main hospitals in Catania. Neonatal accesses are in number significantly lower than Pediatric ones.

We observed that in the three hospitals, we had an important decrease of the total number of pediatric accesses during the SARS-CoV2 pandemic, with a reduction of more than 50% of accesses observed before the pandemic (Fig. 1A).

Neonates who had access to PEU during the study period were 812 in the OC1, 3720 in the OC2, and 748 in the OC3 respectively; 69 (8.4%), 138 (3.7%), and 55 (7.4%) was the specific number and related proportion of neonatal accesses for neurologic conditions (Fig. 1B).

When we analyzed the triage-colored codes of neonates with neurologic symptoms having access to PEU, we found that from 2015 to 2019 the higher proportion of triage color was yellow (60% of all neurologic accesses), while in 2020, during the SARS-CoV2 pandemic, the 67% of neonatal neurologic accesses received a green code.

We observed that the number of neonatal accesses is significantly lower than that observed in pediatric age; nevertheless, if in the OC2 we had an important decrease of neonatal accesses during the SARS-CoV2 pandemic, in the OC3 this decrease was less evident, while in the OC1 the proportion of neonates who had access to PEU remained constant along the study period (Fig. 1B).

**Figure 2:**
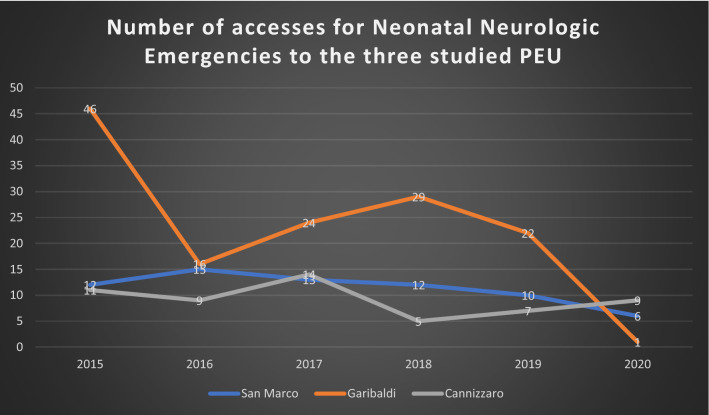
Number of accesses for neonatal neurologic emergencies to the three studied PEU. We can notice that even if the total number of accesses had a drastic decrease during the SARS-CoV2 pandemic period, the number of accesses for neonatal neurologic emergencies in San Marco Hospital remained stable, considering that this hospital is orientated towards the diagnosis and treatment of neonatal neurologic diseases.

Interestingly, we observed that even if during the study period the three hospitals had an important decrease of pediatric accesses to the emergency setting, the proportion of neonates who had access to the OC1 for neurologic diseases, with respect to the total neonatal accesses, remained constantly higher than that one observed in the other two hospitals (Fig. 2).

**Figure 3:**
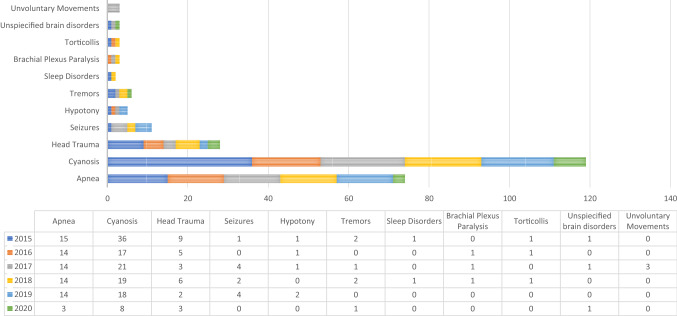
Distribution of accesses according to the neonatal neurologic diseases requiring access to PEU. We can notice that Cyanosis and Apnea were the two more frequent causes for access to PEU in this age group, followed by head trauma and seizures.

In our study, we found that the most frequent neurologic disease for which newborns had access to PEU was Cyanosis, with 121 recorded patients (46.1% of all neonatal accesses for neurologic disease to the three hospitals). Apnea was the second most frequent cause, with a number of 76 accesses (29%), followed by Head Trauma [28 accesses (10.68%)], Convulsions [11 accesses (4.19%)], and Tremors [6 accesses (2.29%)]. Nevertheless, we have to mention that cyanosis and apnea were suspected to be of central origin, because no signs or symptoms were diriment of respiratory disease associated with. Other neurologic causes for which neonates referred to the PEU of the three hospitals were: hypotony, sleep disorders, brachial plexus paralysis, torticollis, unspecific brain disorders, and unvoluntary movements, which accounted for the remaining 7.74% of neonatal accesses for neurologic emergencies (Fig. 3).

## Discussion

The main result of our study showed that the number of neonates having access to PEU is significantly lower than the number of Pediatric patients. Nevertheless, parents have the perception that neurologic symptoms represent an acute emergency that need prompt first aid, above all in neonatal age. In fact, the accesses for neonatal neurologic symptoms and signs remained stable during the study period, above all in the OC1 that is orientated towards the management of neurologic diseases of the infant.

We can suggest that this lower number of neonatal accesses to PEU with respect to the pediatric ones may be associated with the introduction of the Neonatal Emergency Transport Service (NETS) in Sicily, that was firstly activated in 2016. NETS connects Neonatal Intensive Care Units with peripheral residential birth centers, bypassing the PEU first aid filter. The implementation of NETS has played a major role in reducing the neonatal mortality rate in Italy in the last two decades^5,6^, and in reducing neonatal accesses to PEU^7^. This last topic has also been confirmed by a USA study, which showed that the most of neonatal transports are performed by members of the California Perinatal Transport System (CPeTS), carrying critically ill infants from home or peripheral birth centers to NICUs with a higher level of care^7^. Therefore, it seems that the presence of specialized transports for acute neonatal diseases allows an easier access to tertiary care centers, bypassing the PEU first aid filter and facilities^7^.

Interestingly, in our study we found that if on one hand the total number of pediatric accesses in the three hospitals had an important decrease during the SARS-CoV2 pandemic, the number of neonates who had access to PEU for neurologic problems remained stable during the study period, above all in the OC1. This opposite trend might be explained by the fact that parents perceive neurologic symptoms in neonatal age as a medical emergency. Therefore, they need immediate reassurance about the health condition of their infant.

This data could be explained considering that the OC1 is likely more oriented towards the management of neonatal neurologic diseases with specific, targeted, and dedicated paths. Therefore, it is common sense of people in the area to orientate their access towards a neurologic center, rather than to a general PEU, when a neurologic emergency is identified.

Neuro NICU is a special intensive care service in our hospital (OC1). It provides specialized brain-focused care for babies born with a neurologic condition and for babies at risk for brain damages. The Neuro NICU team supports neonates, who have a neurologic disease diagnosed before birth (prenatally), who develop a neurologic problem during delivery, and those who develop a neurologic problem after birth, such as babies who have complications from premature birth^8^.

The establishment of Neonatal Neurocritical Intensive Care Units is supposed to improve neurocognitive long-term outcomes in high-risk patients such as extremely preterm and extremely low birth-weight infants. Dedicated neurocritical care units result by the close cooperation effort between neonatologists and pediatric neurologists^9^.

As far as the analysis of neonatal neurologic diseases that have access to PEU is concerned, Brousseu et al. resumed the neurological causes of mental status alterations and, in particular, of neonatal neurological emergencies, by the acronym “THE MISFITS”^10^. This acronym resumes the major causes for neonatal neurologic emergencies in the following ones: **T**rauma which can be accidental but also not accidental (shaken baby syndrome); **H**ypovolemia; **E**ndocrinological causes (such as congenital adrenal hyperplasia and the condition of thyrotoxicosis); **M**etabolic insufficiency ranging from metabolic decompensation to electrolyte imbalance and acid–base alterations; **I**nborn errors of metabolism; **S**epsis (including meningitis, pneumonia, and urinary tract infection); **F**ormula accidents like under or over dilution; **I**ntestinal events (volvulus, intussusception, and necrotizing enterocolitis); **T**oxins and poisons determining neurological alterations; **S**eizures^10^. Neonatal subtle seizures include: abnormal eye movements (usually horizontal, sustained eye deviation), lip smacking, abnormal tongue movements, pedaling, or apnea^11^.

Among the abovementioned causes, seizures are the most frequently described reason for neonatal access to PEU^12^. Convulsive symptoms occur very often in the first 28 days of life compared to other ages. The true incidence of seizures in neonates is challenging to discern because most population-based studies are based on clinical diagnosis of neonatal seizures, without EEG confirmation. The overall incidence is reported as 1–4 per 1000 live births, with much higher risk among preterm and low birth-weight neonates^12^. The 42–59% of infants who had neonatal seizures show residual abnormalities on neurological examination with poor long-term prognosis depending on the underlying cause and developmental delay is present in over 55% of cases^13^.

Differently to what described in the literature, in our study we found that the most frequent neurological cases of neonatal accesses to PEU in Catania were cyanosis and apnea. Nevertheless, we have to mention that cyanosis and apnea were presumed to be of central origins because no other signs or symptoms were diriment of respiratory disease associated with. Surely, a proper diagnosis should be performed with a cardiorespiratory polygraphy, that is not possible to perform in a Pediatric Acute Setting. However, the absence of other respiratory signs or symptoms, and the negative result of chest X-Ray exams, made us suspect a central origin.

Cyanosis is defined by bluish discoloration of the skin and mucosa. It is a clinical manifestation of desaturation of arterial or capillary blood and could indicate serious hemodynamic as well as cerebral abnormalities^14^. In contrast to acrocyanosis, central cyanosis is visible in the whole body, and is evident in the mucous membranes and tongue. Central cyanosis indicates the presence of potentially serious and life-threatening disease, and requires an immediate evaluation so that the clinician have to rapidly consider and find the respiratory, central nervous system, hematologic, cardiac, and metabolic underlying causes^15^.

Apnea represents the first sign of several neurologic and non-neurologic disorders. Seizure is a relatively underlying infrequent cause of apnea^16^. The Task Force of the American Academy of Sleep Medicine (AASM) defined central apnea as a drop of the airflow for at least 2 respiratory cycles or longer than 20 s, without thoracic effort, associated with a drop in the peak signal excursion by ≥ 90% of the pre-event baseline^17^. Apnoea can also be considered one of the first sign of neurologic disorders. During neonatal period, we can list three forms of apnoea: (1) Central apnoea mentioned and defined before; (2) Ictal Apnea (IA), characterized by the association of apnoeic episodes with cortical EEG activation; (3) Autonomic Apnoea, characterized by activation of the autonomic nervous system and without EEG correlation^18^.

In the literature, some cases of neonatal apnea are described as the only manifestation of seizure, possibly associated with heart rate changes and without a clear positive ictal EEG^19^. This could be linked to the fact that both the degree of myelination and the cortical organization important for the propagation of the electrical activity during seizures are not present or not still mature in neonates, above in preterm babies^19^. In regards, studies on the development of the functional wiring in the neonatal brain showed that myelination and pruning of the synapse terminals occur during postnatal life^20^. However, it seems that cortico-subcortical and vice versa, and most large cortico-cortical networks are drawn before 36 weeks of gestation^20^. Therefore, in case of accesses to PEU for neonatal apnea is extremely important to establish whether a neurologic subtending origin may be the cause.

The differential diagnosis between apnea caused by neurologic diseases and respiratory apnea represents a challenge for neonatologists, as neonatal seizures show more often an electro-clinical impairment, with negative EEG patterns. This might be more challenging when neonatal seizures manifest with only autonomic signs^19^. For this reason, long-term EEG studies integrated with polygraphy Video-EEG are mandatory to detect seizures causing apnea in the neonatal epoque.

Nevertheless, performing diagnosing tests, such as long-term Video-EEG, in an acute setting is not easily practical, and recognition of neurologic subtending emergencies of non-specific symptoms is not always possible. In fact, in our study 90% of patients had to be admitted to NICU for further diagnostic follow-up.

## Limits of the study

In our study we have not included the long-term follow up of these patients, therefore, we do not have the definitive diagnosis of symptoms assessed in PEU.

Long-term follow-up are necessary to determine the subtending diagnosis requiring an emergency access to PEU in the neonatal age.

We moreover do not have data on the diagnostic pathway that these neonates underwent after admission to NICU. A second study with these data will be published following this first preliminary paper.

## Conclusions

Newborns can access to PEU with a variety of signs, symptoms, and behaviors, that may worry their parents, but they also may be normal according to their age. PEU physicians need to be familiar with these normal variations of newborn findings, but also with sign and symptoms of pathological conditions in this age group, in order to distinguish potentially dangerous diseases or appropriately reassure parents when life-threatening events are not present.

If in the literature there are numerous studies on the assessment of diseases that most frequently concern the pediatric or adult patient in an emergency room, there are very few references on neonatal accesses to PEU for critical and urgent neurologic causes. Therefore, appropriate training is required to avoid unnecessary tests without overlooking potentially serious conditions. Although neonates represent a subgroup of patients, it is necessary to establish a diagnostic path, in order to express a rapid judgment on the emergency of the patient during the same initial in order to reduce the risks.

## Data Availability

The datasets generated and/or analysed during the current study are not publicly available due privacy policies but are available from the corresponding author on reasonable request.
